# Loss of nucleosome remodelers CHRAC/ACF does not sensitize early *Drosophila* embryos to X-rays

**DOI:** 10.17912/micropub.biology.000287

**Published:** 2020-07-30

**Authors:** Alessandro Scacchetti, Peter B. Becker

**Affiliations:** 1 Molecular Biology Division, Biomedical Center, Ludwig-Maximilians-University, Munich, Germany

**Figure 1.**
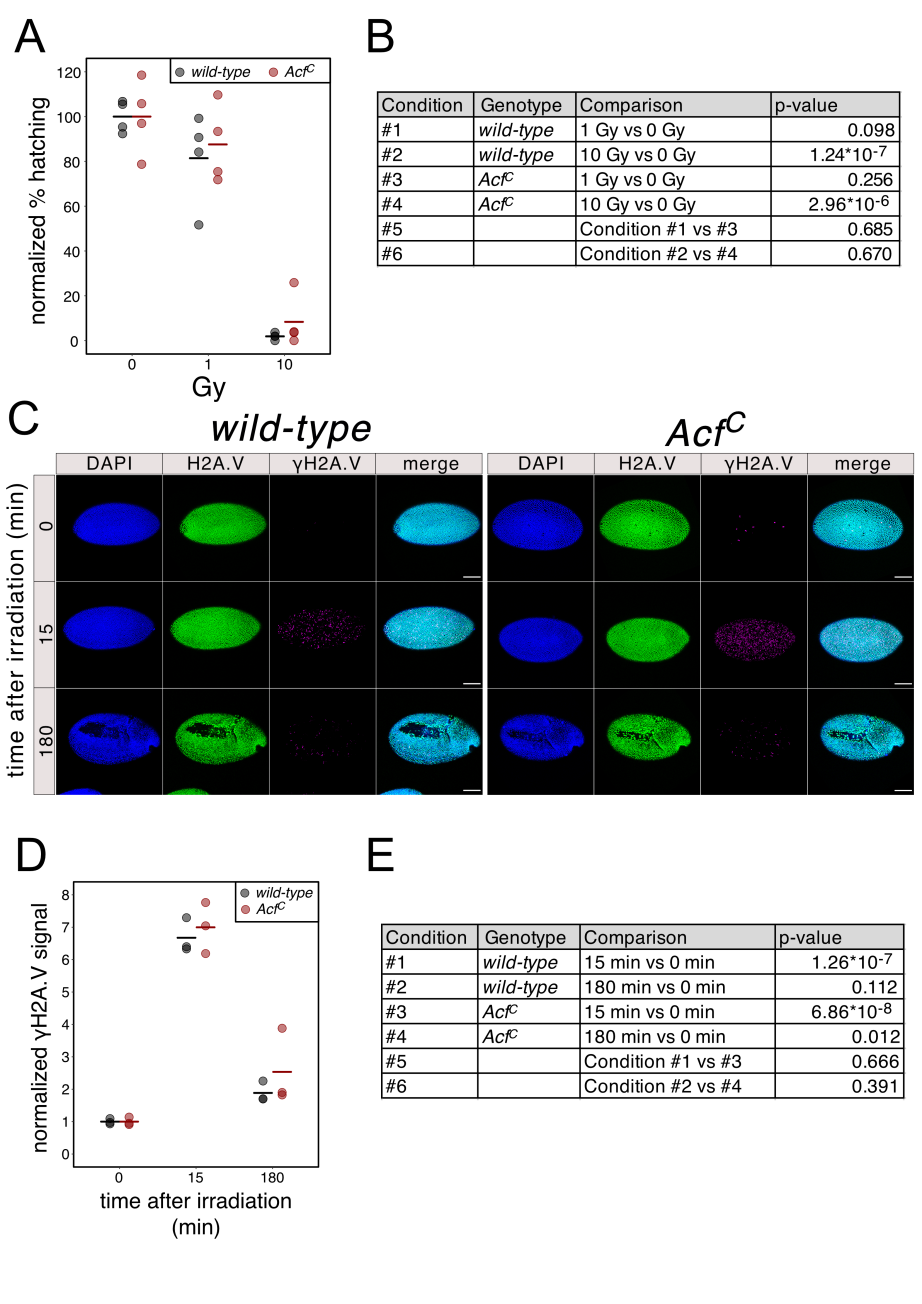
**A.** Effect of X-rays on larval hatching rate. W*ild-type* and *Acf^C^* embryos were irradiated with different X-ray doses (Gy). Each dot represents the % of embryos that hatched in a single experiment (N between 50 and 60 embryos). Horizontal bars represent the mean of the 4 biological replicates. **B.** Calculated p-values (null hypothesis: difference between means = 0) for the comparisons depicted in panel (A). **C.** Representative immunofluorescence images of irradiated (1 Gy) *wild-type* and *Acf^C^* embryos 0, 15 and 180 minutes after irradiation. Embryos were stained with DAPI and with antibodies against H2A.V and γH2A.V. Scale bar = 100 μm. **D.** Quantification of immunofluorescence images of *wild-type* and *Acf^C^* embryos from the time course after X-ray exposure as described in (C). Each dot represents the normalized γH2A.V signal (see methods) of one experiment (N between 8 and 15 embryos). Horizontal bars represent the mean of 3 biological replicates. **E.** Calculated p-values (null hypothesis: difference between means = 0) for the comparisons depicted in panel (D).

## Description

The ‘Chromatin Accessibility Complex’ (CHRAC) and ‘ATP-utilizing chromatin assembly and remodeling factor’ (ACF) of *Drosophila melanogaster* are chromatin remodeling complexes that slide nucleosomes (Becker and Horz, 2002). Both originate from the association of the ATPase ISWI and a large subunit ACF1. CHRAC contains two additional histone-fold subunits, CHRAC-14 and CHRAC-16 (Corona *et al.*, 2000), but its nucleosome sliding activity is essentially similar to ACF *in vitro* (Hartlepp *et al.*, 2005). CHRAC/ACF are implicated in several developmental processes. Mutations of ACF1, which disrupt both complexes, mildly perturb embryogenesis and oogenesis (Boerner *et al.*, 2016; Chioda *et al.*, 2010), and compromise Polycomb silencing and heterochromatin formation (Fyodorov *et al.*, 2004). Through their nucleosome sliding activity CHRAC/ACF contribute to the genome-wide formation of regular nucleosome arrays. Altered nucleosome spacing and regularity in their absence correlates with leakiness of the repressive ground state of chromatin in early embryos (Baldi *et al.*, 2018; Scacchetti *et al.*, 2018). CHRAC/ACF complexes are also conserved in yeast (Iida and Araki, 2004) and mammals (LeRoy *et al.*, 2000; Poot *et al.*, 2000).

CHRAC/ACF may be involved in DNA damage response and repair pathways. The chromatin around a DNA lesion or break must be rendered accessible to the machineries dedicated to the damage recognition and repair (Jeggo *et al.*, 2017). Nucleosome sliders may be involved in freeing the broken DNA from nucleosomes or restoring chromatin after DNA is repaired (Jeggo *et al.*, 2017; Rother and van Attikum, 2017). Human CHRAC/ACF complexes are recruited to DNA breaks and cells depleted of ISWI (SNF2H) or ACF1 (BAZ1A) are more sensitive to DNA damage (Aydin *et al.*, 2014; Klement *et al.*, 2014; Lan *et al.*, 2010; Sanchez-Molina *et al.*, 2011). ACF1 mutant flies show defects in incorporation of the histone variant H2A.V in the *Drosophila* blastoderm stage (Chioda *et al.*, 2010). As in the case of the mammalian H2A.X variant, H2A.V is a central player during the DNA damage response, as its phosphorylation (referred to as γH2A.V) is a key event within damage-triggered signaling cascades (Baldi and Becker, 2013). Due to the high degree of evolutionary conservation of metazoan CHRAC/ACF complexes it is possible that CHRAC/ACF in *Drosophila* are also involved in the DNA damage response, but this hypothesis has not been rigorously tested.

We recently generated an ACF1 loss-of-function allele (*Acf^C^*) by CRISPR-mediated deletion of the *Acf* gene (Scacchetti *et al.*, 2018). To test if this deletion alters the sensitivity of early embryos to DNA damage, we exposed 2-3.5 hour old *wild-type* and *Acf^C^* embryos to two different X-ray doses and measured the rate with which they hatched into larvae. For both genotypes, at a dose of 1 Gy most of the embryos successfully completed embryogenesis (average hatching rates of 81.4% and 87.6% for *wild-type* and *Acf^C^*, respectively) while at 10 Gy only very few of them survive (average hatching rates of 1.9% and 8.3% for *wild-type* and *Acf^C^*, respectively) (**Figure 1A**). We could not measure any significant difference in hatching rates in the *Acf^C^* line (**Figure 1B**) suggesting that ACF1-containing remodelers do not influence the ability of early embryos to cope with DNA breaks.

We also assessed the effect of *Acf* deletion on the immediate early response of chromatin to DNA breakage, the phosphorylation of H2A.V (γH2A.V), which may reveal kinetic differences in DNA damage signaling and repair. Once again, 2-3.5 hour old embryos were exposed to X-rays and fixed for immunofluorescence microscopy 15 min or 3 hours after irradiation. As expected, upon irradiation with 1 Gy a significant increase in γH2A.V signal was observed after 15 min for both genotypes (average signal increase of 6.7 or 7.0 fold for *wild-type* and *Acf^C^*, respectively) (**Figure 1C, D, E**). Three hours after X-irradiation, the γH2A.V signal was reduced (average signal increase of 1.9 and 2.6 fold for *wild-type* and *Acf^C^*, respectively) (**Figure 1C, D, E**), reflecting successful DNA repair. Careful quantification followed by statistical analysis did not show a significant difference of γH2A.V signal between *wild-type* and *Acf^C^* genotypes at both time points, suggesting that the γH2A.V appearance and turnover are unaffected by the absence of ACF1 (**Figure 1E**).

In conclusion, we did not find an effect of CHRAC/ACF depletion on overall DNA damage signaling or developmental competency upon X-ray irradiation in early fly embryos, where ACF1 expression peaks. During this developmental phase, blastoderm cells divide most rapidly, skipping the G1 phase between mitosis and DNA replication [reviewed in (Farrell and O’Farrell, 2014)]. Given this cell cycle peculiarity, our conclusions may not apply to later developmental stages, specific tissues or other developmental processes, such as oogenesis. It is also possible ACF1-containing remodelers play a role in the DNA damage response, but the effect is not obvious because the deficiency is compensated by other remodelers, such as the related RSF complex, which can also slide nucleosomes and may be involved in H2A.V turnover (Hanai *et al.*, 2008).

## Methods

*Acf^C^* mutant flies, backcrossed into *OrR* (*wild-type*), were generated and characterized before (Baldi *et al.*, 2018; Scacchetti *et al.*, 2018). The *Acf^C^* stock is maintained homozygous and all the embryos were collected from crosses of homozygous *Acf^C ^*mothers and fathers. For larval hatching analysis, 2-3.5 h old embryos were collected and placed on agar-apple juice plates. Embryos where irradiated with 0 Gy, 1 Gy or 10 Gy using a Faxitron CellRad X-ray source (130 kV, 5 mA) and incubated for additional 23 h at 25°C. Hatching rate was determined by counting the number of embryos that hatched into larvae. Rates were normalized to the mean of the unirradiated (0 Gy) condition. Plots were generated in R using *ggplot2.* p-values were calculated by fitting a linear model using the *lm* function in R. For immunofluorescence microscopy, 2-3.5 h old embryos on 10 cm collection plates where irradiated with 1 Gy as above. Unirradiated control embryos (0 Gy) were processed along-side. 15 min after irradiation, approximately half of the embryos were collected (15 min time point) from each plate, dechorionated in 25% bleach for 3-5 min and immediately fixed (see below). The remaining ones where kept at 25°C for additional 3 h (180 min time point) and then processed using the same protocol. Unirradiated embryos of the 15 min time point define the ‘0 min’ time point. After extensive washes with water, embryos were transferred to 1.5 ml tubes. Dechorionated embryos were heat-fixed by adding 500 µl of boiling TN solution (0.03% Triton-X-100, 68 mM NaCl) and immediately cooled down by adding 500 µl of ice-cold TN solution and by incubating them on ice for 5 min. The TN solution was replaced by 500 µl of n-heptane and 500 µl of 100% methanol was added. Tubes were shaken for 15 sec and embryos were allowed to settle on ice for 5 min. Embryos were washed twice with 500 µl of methanol and stored at -20°C in 200 µl of methanol. For immunostaining embryos were transferred into 0.2 ml PCR tubes and rehydrated by three 10 min washes with PBS/0.1% Triton-X-100. Embryos were blocked for 3 h in Blocking Solution [PBS/ 0.3% Triton-X-100/ 5% Normal Donkey Serum (Jackson Immuno Research)/ 5% non-fat milk]. After a brief wash with PBS, embryos were incubated overnight at 4°C with primary antibodies diluted in Blocking Solution. Primary antibodies used were rabbit α-H2A.V (1:100) (Börner and Becker, 2016) and mouse α-γH2A.V (1:1000) (UNC93-5.2.1, Developmental Studies Hybridoma Bank) (Lake *et al.*, 2013). Embryos were washed 4 times (15 min each) with PBS/ 0.1% Triton-X-100, then incubated for 3 h at room temperature with secondary antibodies diluted in Blocking Solution (1:300 donkey α-Rabbit.Alexa488, 1:500 donkey α-Mouse.Cy3, both from Jackson ImmunoResearch). Embryos were washed 4 times (15 min each) with PBS/ 0.1% Triton-X-100, then stained with 1:500 DAPI (in PBS) for 10 min. Embryos were washed 2 times (10 min each) with PBS, then mounted using Vectashield (Vector Laboratories).

Pictures were taken using a Leica Sp5 confocal microscope. For quantification, surface pictures of whole embryos were taken using a 20X objective and a 1.27X zoom factor (1024X1024 pixels) maintaining the same settings across all conditions and biological replicates. H2A.V and γH2A.V signals were quantified using a custom script with Fiji (Schindelin *et al.*, 2012). The γH2A.V signal was calculated as follows. For each image the ratio of γH2A.V/H2A.V signal was calculated (“adjusted γH2A.V signal”) and averaged across embryos of the same biological replicate. Then, the adjusted γH2A.V signal for each time point was normalized to the mean of the “0 min” time point. Plots were generated in R using *ggplot2.* p-values were calculated by fitting a linear model using the *lm* function in R.

Fly strains, antibodies, scripts and raw data are available upon request.
